# Supplementary material for: MapperPlus: Agnostic clustering of high-dimension data for precision medicine
Source: PLOS Digit Health. 2023 Aug 9;2(8):e0000307. doi: 10.1371/journal.pdig.0000307 (PMC10411786; doi:10.1371/journal.pdig.0000307)
Supplement: S1 Text — (PDF) [file pdig.0000307.s001.pdf]

Supplementary Material

Modularity

The modularity score, as defined by Newman ([1]), is the most widely accepted quantitative measure of community structure in networks. It is defined as

$$M = \sum_i \left(e_{ii} - a_i^2 \right), \tag{1}$$

where e_{ii} is the fraction of edges that fall within community i and a_i is the fraction of all ends of edges that are attached to vertices in community i. This measure ranges between -1 and 1, with higher values indicating a stronger community structure. A large modularity score suggests that the identified communities of the network are well separated, while a score of zero indicates that the communities are randomly selected.

MapperPlus details

In Step 3, a clustering algorithm is used to cluster the inverse image $f^{-1}(C_n)$ of each hypercube. We store the number of observations within each cluster in the matrix M where $M_{ij} = |i \in P_j|$ where $\mathcal{P} = \{P_j\}$ is the set of all clusters generated by Mapper.

In Step 4 of the process, the Mapper graph G_M is generated using r^m overlapping hypercubes $\mathcal{C} = \{C_j\}$ where m is the dimension of the lensed space. We store the number of observations i (out of a total of N) whose image under the lens f falls into each hypercube in the matrix $H_{N \times r^n}$ where $H_{in} = |i \in C_n|$.

The adjacency matrix of the Mapper graph G_M is defined as $A_M = M^T M$. The edge weights $A_{M_{ij}}$ indicates the number of observations shared between clusters P_i and P_j. Note that by construction, nodes in A_M are connected to themselves.

The adjacency matrix for the graph of instances G is defined as

$$(A_{I_{ij}}) = (MM^T)_{ij} + \sum_{k \in \mathcal{C}} \frac{H_{ik} H_{jk}}{\sum_a H_{ak}}.$$

The term $(MM^T)_{ij}$ denotes the number of nodes in the Mapper graph that contain both observations i and j. The second term sums over all hypercubes that contain both observations i and j and is normalized by the number of number of observations in the hypercube.

Stem Cell Transplant Data Processing

The dataset contained 77 missing values. Given that these datapoints comprised a small fraction of the overall dataset (roughly 1.1% of the observations), these points were replaced with zeroes for the MapperPlus analysis. This is because, given the choice of lens, the zero value would not have a significant effect on output. However, for the statistical testing of the resultant clusters, all missing values were replaced with the mean value of the variable to prevent skewing the results.

The dataset also contained features with missing labelling in the data dictionary. For the sake of completeness, these features were still included in the clustering but not in the summary statistics.

The dataset was normalized (convert to z-scores) prior to analysis.

Numerical Validation MapperPlus Inputs

For all datasets, the metric was Euclidean distance, the lens was the first 2 PCA components, and the clusterer was k-means with 2 clusters. The choice of clusterer was a practical consideration, as the agnostic techniques available generated large numbers of clusters each containing very few observations. We selected 2 clusters for k-means to provide the minimal separation within each hypercube.

For the Wine dataset, the resolution was 7 and gain was 0.7. For the breast cancer dataset, the resolution was 4 and the gain was 0.7. For the iris dataset, the resolution was 4 and the gain was 0.8. For the rice dataset, the resolution was 4 and the gain was 0.7. These resolutions were selected by manual tuning.

Using MapperPlus to predict survival in pediatric transplant patients: MapperPlus inputs

For this analysis, the Euclidean metric was used. We applied a 2-dimensional lens consisting of an Isolation Forest score and the first PCA component. For the choice of clusterer, we

applied k-means with 2 clusters. This choice of clusterer is unusual in that it requires the specification of the number of clusters. We chose 2 clusters because, in the absence of knowing how many clusters may occur, this provides a minimal amount of separation. While agnostic clusterers were available, these methods often yielded arbitrarily large numbers of clusters with too few observations to analyze statistically.

Cluster Validity

For our analysis, we determined that an average NMI score of 0.6 was appropriate. We selected resolution and gain pairs that surpassed this NMI score. It should be noted that we are not selecting the highest possible NMI score. Often, very high NMI scores are indicative of a trivial result. For instance, this can occur at low resolution and high gain, when the majority of observations fall in the overlap between a few hypercubes. In such a scenario, the clusterer is no longer performing a partial clustering, but rather is affecting almost every observation. Then, the stability of the result is completely driven by the clusterer. This is no longer a topological clustering based on Mapper, but a clustering driven by the choice

of clusterer. As such, maximizing NMI is not an appropriate approach, especially when dealing with extreme resolution and gain values. Rather, it is preferred to identify a range of resolution and gain pairs that meet the NMI threshold and perform a manual tuning from that point on.

References

[1] J NME. Fast algorithm for detecting community structure in networks. Phys Rev E. 2004;69:066133. doi:10.1103/PhysRevE.69.066133.
