# Supplementary material for: MapperPlus: Agnostic clustering of high-dimension data for precision medicine
Source: PLOS Digit Health. 2023 Aug 9;2(8):e0000307. doi: 10.1371/journal.pdig.0000307 (PMC10411786; doi:10.1371/journal.pdig.0000307)
Supplement: Attachment — Submitted filename: Datta_ResponseToReviewers.pdf [file pdig.0000307.s003.pdf]

**General response to Reviewers and Editors**

We deeply appreciate the thoughtful and insightful comments of three Reviewers. In light of your comments, we have modified the paper significantly in the following major aspects (specific changes addressed below): we have modified the introduction and literature review to be more thorough and include direct citations to existing research; we have rewritten the discussion to emphasize the contributions of our new method and the wider impact of our work; and we have emphasized patient stratification as the specific aspect of precision medicine our work addresses.

Accordingly, the revision entails a great deal of additions. The introduction is much longer and directly addresses the open questions regarding patient stratification and the role of Mapper. Topological data analysis (TDA) is thoroughly defined and contextualized in the realm of precision medicine. Per Reviewer #1's recommendation, the description of our MapperPlus method has been moved from Results into the Methods section.

Please note that we have updated our central figure slightly: one panel of the flowchart has been edited to better describe the mathematical clustering process that is occurring.

Note: Comments of the Reviewers have been italicized.

**Response to Reviewer #1**

*The term sub-clusters used in the text is confusing. We are talking about clusters, that are homogeneous population sub-groups. We are not dividing clusters to sub-clusters.*

You are absolutely correct that our language was unclear. We have replaced all references to sub-clusters with clusters.

*The literature review starts with the precision medicine requirements and challenges and continues with cluster analysis challenges. Despite that the text in the Introduction part is well constructed and clear, most of the facts and statements are not based on any scientific literature. At least, they are not cited. For example, in the description of 2-nd, 3-rd and 4-th challenges of the use of cluster analysis methods there is not a single citation. The literature review must be significantly expanded. The authors must refer to additional aspects, such as interpretation and multidimensionality in a medical context. Two parts of the Introduction part must be connected in the context of the research topic.*

The lack of citations was a serious mistake in our initial draft and we a thank you for pointing out this obvious lacuna. To address this issue, we have significantly revised the literature review and introduction. First, we have condensed the 4 challenges into 3 key challenges to make our

ideas more concise and direct. To connect the two parts of the introduction, we added a significant portion about the role of Mapper in patient stratification and how that relates to the problem of clustering in a clinical setting. This expanded literature review contextualizes clustering using topological data analysis in the subject of precision medicine. We have also made sure to cite all of our statements.

*In the Materials and Methods section authors must provide the Notations table used in the paper and all databases' characteristics.*

We understand that our use of notation was not clear within the paper. To provide more clarity, all notation used has been defined directly in the line in which it is mentioned, following mathematical convention. Notation that is frequently referred to has been summarized in a table at the start of the Methods section.

*MapperPlus approach must be presented in the Methods section and not in the Results section'*

We agree that the description of the description of the MapperPlus pipeline is more appropriate in the Methods section.

*The authors present the pediatric patients' dataset as a main dataset, but later use 4 different datasets, part of them is not appropriate for the "precision medicine" goal. For example, Iris dataset is not a good example of multivariate data with hidden subpopulations. The Wine dataset has a very small number of independent variables and standard classification techniques provide very high accuracy. The use of cluster analysis approaches is justified when we can assume that there are hidden sub-groups in the population that can be identified by the unsupervised techniques. I suggest using at least 3 multivariate medical datasets with a poor initial classification accuracy.*

We agree that the wine, iris, and rice datasets have no direct clinical relevance but the results we got using them are ultimately critical for the application of MapperPlus in precision medicine. We used these datasets **because** the subpopulations were well-defined. Any unsupervised clustering algorithm must be able to replicate these subpopulations for researchers to have any confidence in the validity of the resulting clusters. We were surprised that the unsupervised clustering algorithms we tested (DBSCAN, Affinity propagation, and Mean Shift) on these datasets produced, in most cases, a number of clusters that were far from the ground-truth numbers as shown in Table 2. As indicated in the manuscript, we tried hard to tune the parameters of DBSCAN to get a high NMI score and to get the number of clusters close to the correct value but were unsuccessful.

Further, we applied MapperPlus to a pediatric stem cell transplant dataset that had no initial classification. MapperPlus found a new set of disjoint clusters whose distinguishing characteristic was patient survival. This is a novel finding of a kind that is important for clinicians to help stratify treatment for their patients.

We would like to point out that we have applied MapperPlus to a dataset of 29 variables from more than 1000 patients admitted to UC Davis Medical Center with acute myocardial infarction. MapperPlus found three clusters of patients that had significantly different survival probabilities. The complexity of this dataset is not atypical of other clinical datasets. The results of this work have been published in abstract form (the reference is below) and we are preparing a full-length manuscript on this work.

Lopez, J., Datta, E., Ballal, A. Izu, L.T. Topological Data Analysis Of Electronic Health Record Features Predicts Major Cardiovascular Outcomes After Revascularization For Acute Myocardial Infarction. AHA Meeting November 2022. Circulation 146, Supplement 1, 14875. Session title: UNDERSTANDING CARDIOVASCULAR RISK: FROM "BIG DATA" TO "REAL WORLD" DATA

*MapperPlus results must be compared to other clustering techniques with the same number of clusters. If the novel method will provide better results, at least in some of the datasets, it can be defined as a successful and usable approach.*

MapperPlus is an agnostic method: it takes no information regarding the number of clusters in a dataset to perform its clustering. Similar methods include affinity propagation, DBSCAN, etc. Many of these methods, like MapperPlus, have tuning parameters, but crucially do not know a priori how many clusters exist in a dataset. As such, when these methods are applied to dataset with n clusters, they may not return n clusters exactly.

In the context of unsupervised learning, it may be difficult to define what a “better” result is, as accuracy cannot be used. We used NMI as a metric to evaluate the adherence of the clustering results to the ground-truth labels. In comparing MapperPlus to the other clusterers listed in Table 3, we found that MapperPlus yielded clusters that more closely matched the ground-truth labels than the other methods. We feel that we have demonstrated that MapperPlus can provide better results in many datasets when compared to similar unsupervised and agnostic clustering techniques.

*The main goal of the research is announced as a novel approach to support the precision medicine. But the main part of the discussion addresses the methodological improvement of the existing clustering methodology. The connection with a medical performance is not emphasized enough.*

We thank you for pointing this out. We completely agree that we did not sufficiently address the implications of MapperPlus for precision medicine. To that end, we more thoroughly discuss the problem of patient stratification and how the existing methodology (which is not a clustering methodology) is insufficient. We then describe how MapperPlus can address the needs of patient stratification.

**Response to Reviewer #2**

*This paper presents an agnostic clustering of high-dimension data for precision medicine. The novelty of this work seems marginal. A clustering method with eight steps is devised. But the significance of the combination of these steps (when compared with the state-of-the-art clustering methods) remains to be justified. Moreover, the precision medicine is a very wide area. It is also unclear for what types of precision medicine data the proposed method is designed.*

Reviewer #2 raises two separate concerns: first, the novelty of our method; and second, what type of precision medicine our work applies to. We will address the latter first.

We completely agree that we did not sufficiently address the implications of MapperPlus for precision medicine. To that end, we more thoroughly discuss the problem of patient stratification and how the widely-used Mapper algorithm (which is not a clustering methodology) is insufficient. We then describe how MapperPlus can address the needs of patient stratification. We relate patient stratification to clustering and discuss how mathematical approaches such as ours can assist with this problem.

Regarding the novelty of our work, we think that our clustering method represents a significant contribution to the literature at the intersection of topological data analysis and precision medicine. Currently, Mapper is widely used, often in patient stratification settings, even though Mapper is not a clustering algorithm. Researchers use ad hoc methods to derive clusters from Mapper, which often require dropping patients from analysis or are not thoroughly described. Thus, there is a lack of a well-defined, rigorously examined method for converting the output of Mapper graphs into disjoint clusters. We provide a novel method that addresses this problem with Mapper, test it on a number of publicly available datasets, and make the software publicly available for researchers. Further, we applied MapperPlus to a pediatric stem cell transplant dataset that had no initial classification. MapperPlus found a new set of disjoint clusters whose distinguishing characteristic was patient survival. This is a novel finding of a kind that is important for clinicians to help stratify treatment for their patients. This has not been done previously. Moreover, this is an important problem in the context of patient stratification, as we wish to identify meaningful patient subgroups from clinical data. As such, our method directly

addresses both a methodological need in the realm of TDA and a technological need in the realm of precision medicine.

**Response to Reviewer #3**

*The main issue is that the description of the study populations is almost absent. There is no description of missing data. Were there missing data, and how did the algorithm deal with that? Or did the researchers exclude missing information (complete analysis), or they applied some imputation technique before the clustering?*

We appreciate this missing information being pointed out. We have revised the manuscript to mention this information. Our dataset was not missing any data points, but we were missing a data dictionary for a few of the categorical variables. As such, we included those categorical variables in our analysis, but we did not include those variables in our final discussion of the results. We discuss the problem of the missing data dictionary in the Discussion.

*It is unclear whether the algorithm normalised the data or whether it should have been normalised before the application.*

We were remiss in not indicating that the data had been normalized. While both normalized and raw data could be inputted into the algorithm, best practices dictate that data should be normalized. We updated the manuscript to include that all data were converted to z-scores.

*The Cluster quality assessment using NMI scores is only possible if the data are labelled. If the pipeline has an agnostic approach (not labelled), how can NMI be applied for selecting parameters?*

NMI is used separately in two places: to assess algorithm performance on labeled data and to select parameters. For the former, we do require data labels. We use NMI to compare the original data labels to the output from MapperPlus (or whichever clustering technique is used) as a measure of the quality of our clustering. This is not how the NMI scheme would be used in practice, but is solely a method for comparing our method to other algorithms.

The use of NMI to select parameters is done as follows. Suppose you select resolution 10, gain 0.8 and this yields a clustering U. Then, you look at another parameter set, say resolution 10, gain 0.9. Since the gain is similar in this case, we would expect the resultant clustering U' to be similar to U. This would be indicative of stability. We use NMI to compare U to U', that is, we compute NMI(U, U'), which yields a score from 0 to 1. A score close to 1 indicates that U and U' share a great deal of information, while a low score indicates very little mutual information. To evaluate the stability at resolution r and gain g, we compute the pairwise NMI score with all

the nearby resolution and gain pairs and average it. This gives us a stability score for the clustering U(r,g). This does not require labeled data, but rather the labeling (or clustering) that MapperPlus yields. We have expounded on this in the manuscript to make the distinction clearer.

*The article describes clusters and subclusters. Is there any difference?*
We thank you very much for pointing our inconsistent use of these two terms. Our revised manuscript uses only the term *cluster*.

*Can the clustering method be parametrised?*
One answer to your question is a very straightforward one: The MapperPlus algorithm requires two parameters, resolution and gain, upon which its results are dependent. However, we suspect you are asking a much deeper question of whether clustering methods could be somehow be mapped to some function. We are intrigued by such a variational approach but we, frankly, have no answer to this question.

*Define what TDA is*
We have revised the introduction to discuss topological data analysis (TDA) in detail and demonstrate how precision medicine can benefit from it.

*Is the lens selection dependent on the hyper-parameters of the algorithm?*
No, as we describe in the manuscript, the lens is simply a function which maps that data to a lower dimensional space. To clarify this, we include a more thorough description of the function in the revised text.
