# Supplementary material for: MapperPlus: Agnostic clustering of high-dimension data for precision medicine
Source: PLOS Digit Health. 2023 Aug 9;2(8):e0000307. doi: 10.1371/journal.pdig.0000307 (PMC10411786; doi:10.1371/journal.pdig.0000307)
Supplement: Attachment — Submitted filename: Review - MapperPlus Agnostic Clustering of High-Dimension Data for Precision Medicine.docx [file pdig.0000307.s002.docx]

**Review**

**MapperPlus Agnostic Clustering of High-Dimension Data for Precision Medicine**

The article deals with a new unsupervised approach to support personalized medicine decisions (precision medicine). The authors suggest using clustering algorithm that assumes low error clustering (agnostic clustering).

Comments:

1) The term sub-clusters used in the text is confusing. We are talking about clusters, that are homogeneous population sub-groups. We are not dividing clusters to sub-clusters.
2) The literature review starts with the precision medicine requirements and challenges and continues with cluster analysis challenges. Despite that the text in the Introduction part is well constructed and clear, most of the facts and statements are not based on any scientific literature. At least, they are not cited. For example, in the description of 2-nd, 3-rd and 4-th challenges of the use of cluster analysis methods there is not a single citation. The literature review must be significantly expanded. The authors must refer to additional aspects, such as interpretation and multidimensionality in a medical context. Two parts of the Introduction part must be connected in the context of the research topic.
3) In the Materials and Methods section authors must provide the Notations table used in the paper and all databases' characteristics.
4) MapperPlus approach must be presented in the Methods section and not in the Results section.
5) The authors present the pediatric patients' dataset as a main dataset, but later use 4 different datasets, part of them is not appropriate for the "precision medicine" goal. For example, Iris dataset is not a good example of multivariate data with hidden subpopulations. The Wine dataset has a very small number of independent variables and standard classification techniques provide very high accuracy. The use of cluster analysis approaches is justified when we can assume that there are hidden sub-groups in the population that can be identified by the unsupervised techniques. I suggest using at lest 3 multivariate medical datasets with a poor initial classification accuracy.
6) MapperPlus results must be compared to other clustering techniques with the same number of clusters. If the novel method will provide better results, at least in some of the datasets, it can be defined as a successful and usable approach.
7) The main goal of the research is announced as a novel approach to support the precision medicine. But the main part of the discussion addresses the methodological improvement of the existing clustering methodology. The connection with a medical performance is not emphasized enough.
