# Supplementary material for: MapperPlus: Agnostic clustering of high-dimension data for precision medicine
Source: PLOS Digit Health. 2023 Aug 9;2(8):e0000307. doi: 10.1371/journal.pdig.0000307 (PMC10411786; doi:10.1371/journal.pdig.0000307)
Supplement: Attachment — Submitted filename: Datta_ResponsetoReviewers.docx [file pdig.0000307.s004.docx]

Dear Dr. Rappoport:

We thank you for guiding our manuscript through the review and we thank the two reviewers for their comments that, we feel, have greatly improved our paper. We have made some small changes that improve the clarity of the paper; these are distinguished by the red color.

Reviewer 1’s concern in both the initial and revised manuscripts was that we did not use an unlabeled dataset. The transplantation dataset is, in fact, unlabeled. To our embarrassment, we now realize that the way the first two versions of the paper was written, the transplantation dataset could be viewed as a labeled. We have rewritten the paper so that it is clear that this dataset is unlabeled. We regret the confusion that our poor writing has caused.

We have included a new paragraph summarizing the MapperPlus algorithm as suggested by Reviewer 2. We appreciate Reviewer 2’s bringing attention to ensemble clustering and the suggestion to consider ensemble clustering in our future extensions of MapperPlus and clustering. After reading the suggested papers, we see that both ensemble clustering and MapperPlus share a similarity in generating an intermediate graph to facilitate the attainment of optimal clustering. However, there is a crucial difference between the two methods: the graph for the ensemble clustering method is most similar to a consensus graph, while the graph generated by MapperPlus (and Mapper) is focused on creating a topological representation of the data that preserves the local connectivity among data points. We explain this in our response to Reviewer 2. We did not introduce ensemble methods into the paper as we thought this might take the paper too far afield.

Responses to Reviewer 1’s comments:

We appreciate the comments you’ve made on both the original and revised manuscripts.  Your comments have, we think, significantly reshaped and improved the paper.  We hope you agree.

(1) The two changes in lines 5 and 10 in the abstract you’ve suggested have been made.  Thank you for catching these mistakes.

(2) We have rewritten a significant portion of the paper to address your main concern of using a dataset that had truly hidden classes (lines 298—310, 460—465 of the marked-up version). To evaluate the performance of MapperPlus and other clustering algorithms we used several publicly available datasets with known labeled clusters. The datasets are wine, iris, rice, and breast cancer. Note that the breast cancer dataset is a medical dataset consisting of cells that are benign or malignant. In actual precision medicine applications, datasets have no labeled clusters.  Indeed, the whole point of running any clustering algorithm is to find clusters in the dataset.

     	To test MapperPlus on a medically relevant dataset that had no labeled cluster and is publicly available, we used the transplantation dataset.  Moreover, this dataset is similar to many medical datasets in having a mix of continuous and categorical data.  We explain why we chose this dataset on lines 298—310 of the marked-up version.

     	In summary, we employed two medical datasets in this study: the breast cancer dataset for validation of our approach and the transplantation dataset to demonstrate a realistic precision medicine application and the clinical relevance of the clusters identified by MapperPlus. We now realize that the way we wrote the original manuscript and the first revision, the transplantation dataset might be construed as having labeled clusters.  This dataset has categorical variables such as acute myeloid leukemia or lymphoma, but we do not consider them as cluster labels as none of them completely characterize the patients.  These categorical variables are unlike, say, Setosa (one of the species in the iris dataset) or the benign/malignant (of the breast cancer dataset) labels that completely characterize the set of observations within a cluster.  We explain why we did not take any of the categorical variables in the transplantation dataset to be a cluster label in lines 303—310 of the marked-up version.  We regret the confusion that our poor writing has caused and we thank you for the time you’ve given to our paper.

Response to Reviewer 2’s comment

We greatly appreciate your feedback, as it has significantly contributed to improving the quality and readability of our paper. We have made the following changes based on your suggestions.

(1)   We have added a paragraph (lines 244—250 of the marked-up version) following the MapperPlus method to provide a concise summary of the importance of each step in MapperPlus. This summary offers a broader outline of the underlying idea behind MapperPlus, enhancing the clarity and coherence of our paper.

(2)   We greatly appreciate you bringing these ensemble clustering algorithms to our attention. They are highly relevant, although there are some differences. While it is important to note that the goals of ensemble clustering and MapperPlus differ, there is a shared concept between the two methods. Both approaches involve the generation of an intermediate graph to facilitate the attainment of optimal clustering. Ensemble methods produce a bipartite graph comprising instances and clusters, similar to the Mapper graph (graph of partial clusters) and the network of instances that is generated from Mapper graph. In ensemble methods, the graph of instances and clusters is utilized to derive the optimal clustering, which closely resembles the process of clustering the Mapper graph and network of instances to obtain disjoint clusters in MapperPlus. The crucial difference is a paradigmatic one: the graph for the ensemble clustering method is most similar to a consensus graph, while the graph generated by MapperPlus (and Mapper) is focused on creating a topological representation of the data that preserves the local connectivity among data points. In future work, we plan to explore the performance of these techniques comparatively.
